# Optimal Configuration of Multi-Task Learning for Autonomous Driving

**DOI:** 10.3390/s23249729

**Published:** 2023-12-09

**Authors:** Woomin Jun, Minjun Son, Jisang Yoo, Sungjin Lee

**Affiliations:** 1 Electronic Engineering, Dong Seoul University, Seongnam 13117, Republic of Korea; 2 Autonomous Driving Lab., MODULABS, Seoul 06252, Republic of Korea

**Keywords:** autonomous driving, multi-task learning, lane detection, object detection, drivable area segmentation, depth estimation

## Abstract

For autonomous driving, it is imperative to perform various high-computation image recognition tasks with high accuracy, utilizing diverse sensors to perceive the surrounding environment. Specifically, cameras are used to perform lane detection, object detection, and segmentation, and, in the absence of lidar, tasks extend to inferring 3D information through depth estimation, 3D object detection, 3D reconstruction, and SLAM. However, accurately processing all these image recognition operations in real-time for autonomous driving under constrained hardware conditions is practically unfeasible. In this study, considering the characteristics of image recognition tasks performed by these sensors and the given hardware conditions, we investigated MTL (multi-task learning), which enables parallel execution of various image recognition tasks to maximize their processing speed, accuracy, and memory efficiency. Particularly, this study analyzes the combinations of image recognition tasks for autonomous driving and proposes the MDO (multi-task decision and optimization) algorithm, consisting of three steps, as a means for optimization. In the initial step, a MTS (multi-task set) is selected to minimize overall latency while meeting minimum accuracy requirements. Subsequently, additional training of the shared backbone and individual subnets is conducted to enhance accuracy with the predefined MTS. Finally, both the shared backbone and each subnet undergo compression while maintaining the already secured accuracy and latency performance. The experimental results indicate that integrated accuracy performance is critically important in the configuration and optimization of MTL, and this integrated accuracy is determined by the ITC (inter-task correlation). The MDO algorithm was designed to consider these characteristics and construct multi-task sets with tasks that exhibit high ITC. Furthermore, the implementation of the proposed MDO algorithm, coupled with additional SSL (semi-supervised learning) based training, resulted in a significant performance enhancement. This advancement manifested as approximately a 12% increase in object detection mAP performance, a 15% improvement in lane detection accuracy, and a 27% reduction in latency, surpassing the results of previous three-task learning techniques like YOLOP and HybridNet.

## 1. Introduction

Recent technical innovations in deep learning have led to a quantum leap in robot technology and autonomous driving technology [1]. In particular, various sensors, such as cameras, lidar, radar, GPS, ultrasonic waves, and IMUs, are used to acquire and process diverse information related to vehicle situational awareness in order to make driving judgments and control the vehicle [1,2,3].

However, to apply the information gathered from these various sensors to autonomous driving in real time, the corresponding calculations must be lightweight and accelerated [4,5,6,7,8,9,10,11,12,13,14,15,16,17,18]. Among these sensors, the tasks that require the highest computation and latency are 2D and 3D context-aware computations, which primarily involve cameras, lidar, and radar. In studies [6,7,8,13,14,15], network weight reduction and acceleration efforts were conducted for camera-based 2D object detection and 2D segmentation calculations. In studies [5,14,15], quantization, pruning, and knowledge distillation methods for light weighting of deep learning were studied. Study [18] performs acceleration research for camera-based lane detection.

However, because all these studies focus on single tasks, the corresponding operations must be combined in a real environment where all of them must be used. For this reason, research on MTL (multi-task learning) was initiated in studies [9,10,11,12,19,20,21], allowing multiple tasks noted above to be performed simultaneously as much as possible. Multi-task learning (MTL) is a learning paradigm in machine learning and its aim is to leverage useful information contained in multiple related tasks to help improve the generalization performance of all the tasks [22]. Therefore, due to the parallel execution characteristics of multi-task learning (MTL), essential image recognition tasks for autonomous driving, such as 2D object detection, lane detection, and drivable area segmentation, were conducted using MTL. However, among these studies on multi-task learning, there has been no research addressing optimal design methodologies for three-task configurations. In fact, most multi-task learning (MTL) studies feature complex structures and intricate training processes, making it challenging to reproduce their performance. Particularly in multi-task learning (MTL), the determination of an optimal combination of components is critical. This includes the shared backbone and its lightweight version, subnets for each task, loss functions dictating subnet training performance, and task-specific optimizers and training details, all of which significantly impact the safety of autonomous driving. From an accuracy perspective, concurrently executing multiple tasks can lead to improper training of the shared backbone weights, potentially degrading each task’s performance and adversely affecting the safety of autonomous driving. In terms of latency, if the latency of each task slows down beyond the required threshold, it can prevent the decision-making and control stages of autonomous driving from being executed within an appropriate time frame, leading to potentially severe accidents. Additionally, regarding memory size, if each task consumes an increasing proportion of the limited hardware memory in an autonomous vehicle, it can place additional load on the overall system operations, compromising stability. Therefore, in this study, we experimented with various combinations of these details that determine the performance of each task in MTL and proposed a solution through the MDO (multi-task decision and optimization) algorithm to find the optimal configuration.

## 2. Related Work

In the field of situational awareness for self-driving technology, it is crucial to execute image recognition tasks with high precision in real time. In particular, information from various sensors should be utilized to enable safe and reliable driving decisions.

Among these, representative image recognition tasks based on cameras include 2D tasks such as object detection, semantic segmentation, and lane detection, as well as 3D tasks such as 3D object detection and 3D segmentation. First, 2D detection studies of [8,23] achieve an accuracy of 52 AP with a performance of over 30 FPS based on a single stage. Recently, anchor free-based technologies, as explored in [24,25], have developed a technique that achieves a performance of 280 FPS or more, while also enhancing accuracy. In the field of 2D semantic segmentation, research studies [26,27] have announced a technology that delivers an accuracy performance of 82.4 mAP. In the field of 3D object detection research, studies based on cameras [28] (18.69% AP), lidar [29] (81.8% AP), and a fusion of camera–lidar sensors [30] (82.4% AP) have been announced. In the field of 3D semantic segmentation, lidar-based research [31] has achieved a performance of 74% mIoU. In the studies by [32,33], acceleration of depth estimation was investigated using only cameras, whereas the research conducted by [34,35] focused on exploring camera-based 3D object detection. The research presented in [36,37] dealt with the acceleration of camera-based 3D reconstruction. Lidar-based 3D object detection and 3D segmentation were the subjects of studies by [38,39,40]. Finally, the research by [41,42] investigated radar-based 3D object detection.

However, we need to note that the technologies discussed above pertain to studies of individual tasks. In practice, within an autonomous vehicle, when all the corresponding recognition models are loaded and executed simultaneously, there can be complications due to synchronization issues among various technologies and potential system overloads. In other words, even if only some of the various image recognition tasks in autonomous driving meet the accuracy or latency requirements, but others do not, it can negatively impact the safety of autonomous driving.As a result, the exploration of MTL (multi-task learning) was initiated specifically for autonomous driving applications [19,20,21].

MTL aims to leverage useful information contained within related tasks to enhance the generalization performance of all tasks. MTL can be categorized into five technical approaches based on its characteristics: feature learning approach [43] low-rank approach [44], task clustering approach [45], task relation learning approach [46], and decomposition approach [47]. These approaches are being utilized in various domains of deep learning, including natural language processing [48], reinforcement learning [49], medicine [50], and computer vision [43].

Additionally, in the field of autonomous driving, extensive research is being conducted to improve the performance of related tasks using MTL. In HybridNet, MTL was investigated with respect to three tasks: drivable area segmentation, lane detection, and object detection [19]. Additionally, YOLOP demonstrated potential by enhancing the performance of HybridNet for MTL, focusing on the aforementioned three tasks [20,21].

From the foregoing, it is evident that the accuracy performance of each MTL task is influenced by the efficiency of the underlying backbone network. However, as indicated by the studies referenced in [51,52], the use of a complexly structured backbone network, such as ViT (Vision Transformer) [53], does not necessarily ensure high accuracy across all tasks. This makes achieving the ultimate objective of driving quite challenging. These findings underscore the importance of designing image recognition technology that takes into account the mutually complementary relationship among the relevant technologies.

The principal contributions of this study are as follows:

1. This study proposes an optimal neural network architecture incorporating backbone and loss functions for triple-task learning of drivable area segmentation, object detection, and lane detection. It achieves improvements in all aspects, including accuracy, latency, and size, compared to traditional individual tasks and previous three-task learning methods.
2. The integration of depth estimation within the MTL framework for 2D image recognition was explored, and it was proven to be unsatisfactory due to the low ITC (inter-task correlation).
3. For the performance optimization of MTL, a 3 step MDO algorithm was applied, along with additional training techniques based on SSL (semi-supervised learning). This approach enables enhancements in all aspects, including accuracy, latency, and memory size.

## 3. System Model

### 3.1. MTL Architecture

**Figure 1:**
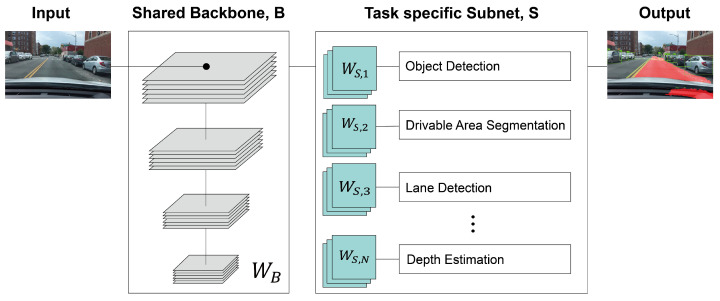
The architecture of multi-task learning for autonomous driving.

Figure 1 presents a system overview of multi-task learning for autonomous driving. As depicted in Figure 1, tasks such as OD (object detection), DAS (drivable area segmentation), LD (lane detection), DE (depth estimation), and others share a common backbone network model SBM (shared backbone model) denoted as B. Subsequently, each individual task utilizes its own dedicated subnet model TSM (task-specific subnet model) denoted as S, along with respective loss functions, to derive the final output. Among the networks based on the encoder–decoder structure, UNet [54], FPN [55], Bi-FPN [56], PFPN [26], and Transformer [27] were selected as candidates for SBM. Because all of these networks are based on segmentation tasks, most of them can be shared for tasks such as object detection, segmentation, and depth estimation. As evident from the above, the essence of MTL lies in sharing a common backbone, which serves as a fundamental module or a subset thereof, across diverse image recognition tasks. By sharing the backbone in this manner, the latency required for each multi-task can be reduced, and the accuracy can also be improved. This is because the shared backbone undergoes learning on diverse data from each multi-task, leading to enhanced performance. However, attempting to share the same backbone for tasks that are too unrelated among these multi-tasks, i.e., low ITC (inter-task correlation), may lead to decreased accuracy for each individual task. This is because the shared backbone cannot be optimized specifically for each task, compromising its performance. In particular, in environments such as self-driving cars, even a minor error in image recognition performance can have a potentially fatal impact on driver safety. Hence, the application of MTL in such scenarios necessitates a cautious approach due to the critical nature of the problem.

### 3.2. MDO Algorithm

**Figure 2:**
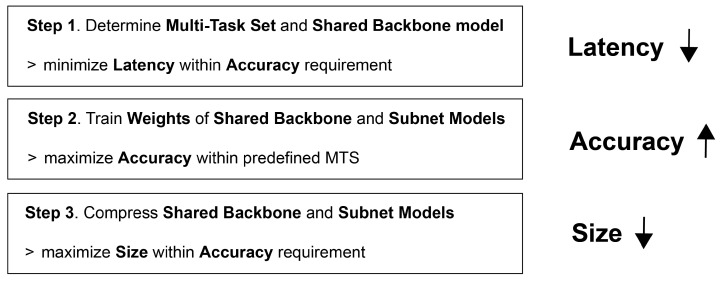
MDO algorithm.

As shown in Figure 2, this paper introduces the MDO (multi-task decision and optimization) algorithm consisting of three steps, designed to optimize accuracy, latency, and size across multiple tasks. The algorithm focuses on minimizing the latency and the size while satisfying the target accuracy considering the target performance of each task. The parameters for the description of the MDO algorithm are defined in Table 1.

- Step 1. Determination of MTS $T_{m}^{*}$ and SBM $B^{*}$ that can minimize total latency with satisfying each accuracy requirement: Based on the weight values pretrained on ImageNet, optimal MTS (multi-task set) $T_{m}^{*}$ and optimal SBM (shared backbone model) $B^{*}$ are determined according to Equation (1). In addition, as optimal MTS $T_{m}^{*}$ is determined, TSM S suitable for $T_{m}^{*}$ is also determined.
  (1) $$\begin{array}{ccc} T_{m}^{*},B^{*} & = & \arg\min_{T_{m},B}\left\{\sum_{t_{i}\in T_{m}}LM\left(t_{i},B\right)+\sum_{t_{j}\in T_{m^{c}}}LI\left(t_{j}\right)\right\}\\ \mathrm{Subject}\;\mathrm{To}\; & & Acc\left(t_{i}\right)\geq \gamma_{i},\;\;t_{i}\in T_{t},\\ & & B\in \{\mathrm{UNet},\mathrm{FPN},\mathrm{BiFPN},\mathrm{PFPN},\mathrm{Transformer}\}. \end{array}$$
  where the parameters are defined in Table 1.
  Equation (1) is formulated to select the optimal shared backbone model $B^{*}$ and the optimal multi-task set $T_{m}^{*}$, aimed at minimizing the latency of the multi-task set within a multi-task learning framework. For this, each task $t_{i}$ within the multi-task set must satisfy the accuracy requirement, and each SBM is chosen from UNet, FPN, BiFPN, PFPN, and Transformer. The weights $W_{B}$ and $W_{S}$, being pretrained on ImageNet, do not require additional training. This allows for a swift check of the accuracy conditions for each task $t_{i}$ in the MTS, thereby selecting the multi-task set that minimizes the overall latency.
  In addition, the accuracy threshold $\gamma_{i}$ in Equation (1) is closely linked to the safety and latency in autonomous driving. It plays a crucial role in shaping the overall operational methodology of multi-task learning. First of all, if the goal is to enhance accuracy through MTL, it can be achieved by introducing a new accuracy threshold, $\gamma_{i}+\delta_{i}$, where $\delta_{i}$ is added to the existing accuracy threshold, $\gamma_{i}$. Certainly, it should be noted that increasing the accuracy threshold in this manner may result in the non-existence of a feasible solution for the MTS $T_{m}$. On the contrary, if the accuracy threshold is decreased to $\gamma_{i}-\delta_{i}$, it becomes possible to obtain/larger MTS $T_{m}$, thereby reducing the overall system latency. However, this reduction in accuracy threshold may negatively impact the safety of autonomous driving. Based on this observation, it becomes evident that determining $T_{m}$ based on Equation (1) introduces a trade-off relationship between safety and speed. Furthermore, because the accuracy threshold $\gamma_{i}$ can be customized individually for each task, it provides the flexibility to prioritize specific tasks over others. In other words, by setting a higher threshold for an image recognition task that directly impacts the safety of autonomous driving and a slightly lower threshold for tasks of lesser importance, a viable solution to the problem can be established. These distinct accuracy thresholds also influence the decision-making process of the SBM $B^{*}$.
- Step 2. Determination of $W_{B}^{*}$ and $W_{S}^{*}$ that can further maximize the accuracy of each task within the determined MTS $T_{m}^{*}$:
  Using the previously determined MTS $T_{m}^{*}$ and SBM $B^{*}$, the optimal weights $W_{B}^{*}$ for the SBM and $W_{S}^{*}$ for the TSM are determined in order to maximize the accuracy across all tasks. Due to the potential variation in accuracy scales and the varying importance of each task, the weights $W_{B}$ and $W_{S}$ are retrained to maximize the total weighted accuracy sum, incorporating task-specific weights $\lambda_{i}$ as depicted in Equation (2),
  (2) $$\begin{array}{ccc} W_{B}^{*},W_{S}^{*} & = & \arg\max_{W_{B},W_{S}}\left\{\sum_{t_{i}\in T_{m}^{*}}\lambda_{i}\cdot Acc\left(t_{i},W_{B},W_{S}\right)\right\}, \end{array}$$
  (3) $$\begin{array}{ccc} & \;\;\approx & \arg\min_{W_{B},W_{S}}\left\{\sum_{t_{i}\in T_{m}^{*}}\lambda_{i}\cdot L\left(t_{i},W_{B},W_{S}\right)\right\}. \end{array}$$
  Here, task-specific weights $\lambda_{i}$ are normalized to a total sum of one, $\sum_{t_{i}\in T_{m}^{*}}\lambda_{i}$, and are proportionally determined for each task based on the respective loss functions, as established through experimentation. Because the accuracy value of Equation (2) can be replaced with a loss function for training, it can be re-derived as in Equation (3) to ensure an optimized allocation of resources across tasks. Additionally, the task-specific loss functions in Equation (3) are detailed in Section 4.
- Step 3. Network compression that can minimize the size of $W_{B}^{*}$ and $W_{S}^{*}$ while satisfying all accuracy and latency requirements: In Step 3, the memory size of the predetermined optimal weights $WB^{*}$ and $WS^{*}$ for the shared backbone model and task specific model is minimized. However, this network compression is conducted in a manner that does not compromise the accuracy and latency values obtained in the previous stage. Based on the study by [5], network compression is performed through quantization and pruning to determine the lightweighted $W_{B}^{}$ and $W_{S}^{}$. This task is carefully managed to minimize network size while maintaining the target accuracy, as it can potentially impact accuracy. Based on the research [5], we conduct network compression through quantization and pruning to determine the lightweighted $W_{B}^{-}$ and $W_{S}^{-}$ from $W_{B}^{*}$, $W_{S}^{*}$,
  (4) $$\begin{array}{ccc} W_{B}^{-},W_{S}^{-} & = & \arg\min_{W_{B}^{*},W_{S}^{*}}\left\{\sum_{t_{i}\in T_{m}^{*}}Size\left(t_{i},W_{B}^{*},W_{S}^{*}\right)\right\},\\ \mathrm{Subject}\;\mathrm{To}\; & & Acc\left(t_{i}\right)\geq \gamma_{i},\;\;t_{i}\in T_{m}^{*},\\ & & LM\left(t_{i},B\right)\geq \theta_{i},\;\;t_{i}\in T_{m}^{*}. \end{array}$$
  According to [5], most quantization techniques rely on a quantization table, which can lead to latency loss due to value reference time. However, FP16, which performs quantization by merely truncating decimal values from the original FP32 values, is the only method that can achieve quantization without latency loss. Pruning can also achieve network compression without impacting latency. However, unlike FP16, pruning requires additional training. Moreover, the accuracy can be compromised depending on the training technique used, thus necessitating careful application of this method. Considering these factors, this study prioritizes the application of FP16, aiming to achieve network compression without sacrificing the accuracy and latency gains achieved in the previous phase.

## 4. Subnet for Multi-Task Learning

In this section, we focus on identifying the TSM (task-specific subnet model) S for MTL. In particular, the neural network structures and loss functions for the mentioned tasks, i.e., object detection, drivable area segmentation, lane detection, and depth estimation are defined.

### 4.1. OD (Object Detection)

For object detection, we employ the multi-head subnet structure of FPN [55] based RetinaNet [8] as a subnet. Additionally, the loss functions used for each multi-head branch are the focal loss and regression loss as

$$\begin{array}{c} L_{OD}=L_{Foc}+L_{Reg}. \end{array}$$

First, focal loss is a cross-entropy-based loss function designed to address the class imbalance problem and is defined as follows:

$$\begin{array}{c} L_{Foc}=-\alpha_{t}\cdot {\left(1-p_{t}\right)}^{\gamma }\cdot \log\left(p_{t}\right), \end{array}$$

where $\alpha_{t}$ is a weighting factor for each class, which helps to balance the importance of positive/negative examples, $p_{t}$ is the model’s estimated probability for the class with label 1 (ground truth), and γ is a focusing parameter. A higher value of γ dampens the loss contribution from easy examples and increases the influence of hard examples. In this paper, $\alpha_{t}$ is equally set to 1/N for all classes, and γ is set to 2.

Regression loss $L_{Reg}$ is a loss function introduced for bounding box regression. It uses Smooth *L*1 loss (Huber loss) and is defined as follows:

$$\begin{array}{c} L_{Reg}=\left\{\begin{array}{cc} 0.5\times {\left(\delta y\right)}^{2} & \mathrm{if}\;|\delta y|<1,\\ |\delta y|-0.5 & \mathrm{otherwise}, \end{array}\right. \end{array}$$

where δy represents the difference between the predicted value and the ground truth for each aspect of the bounding box (e.g., center coordinates, width, and height).

The accuracy index utilizes *AP* (average precision) for each major class and *mAP* (mean average precision) for all classes:

$$\begin{array}{c} AP=\int_{0}^{1}p\left(r\right)dr,\;\;\;\;mAP=\frac{1}{N}\sum_{i=1}^{N}AP_{i}, \end{array}$$

where p(r) is the precision at recall *r*, *N* is the number of classes. and $AP_{i}$ is the *AP* for the *i*th class.

### 4.2. DAS (Drivable Area Segmentation)

For DAS (drivable area segmentation) tasks, the subnet depending on the each semantic segmentation based backbone [26,27,51,54,55,56] is primarily utilized. In terms of the loss function, Dice loss, Tyversky loss, and BCE loss are selectively employed based on their performance.

BCE (binary cross-entropy) loss $L_{BCE}$ is based on pixel-wise classification, that is, each pixel in an image is classified as either belonging to the foreground class or the background class,

$$\begin{array}{c} L_{BCE}=-\frac{1}{N}\sum_{i=1}^{N}\left[y_{i}\log\left(p_{i}\right)+\left(1-y_{i}\right)\log\left(1-p_{i}\right)\right] \end{array}$$

where *N* is the total number of pixels in the image, $y_{i}$ is the ground truth label for the *i*th pixel, which is 1 if the pixel belongs to the foreground and 0 if it belongs to the background, $p_{i}$ is the predicted probability that the *i*th pixel belongs to the foreground class.

The Dice loss $L_{Dice}$ utilizes the DC (Dice coefficient), a measure used to quantify the degree of overlap between two sets. This coefficient computes the extent of overlap between the predicted area and the actual ground truth, normalizing it to a decimal value less than 1. The Dice loss $L_{Dice}$ is then derived by calculating the difference from 1,

$$\begin{array}{c} DC=\frac{2\times |P⋂G|}{\left|P|+|G\right|}=\frac{2\times \sum_{i=1}^{N}p_{i}g_{i}}{\sum_{i=1}^{N}p_{i}^{2}+\sum_{i=1}^{N}g_{i}^{2}},\;\;\;L_{Dice}=1-DC, \end{array}$$

where $p_{i}$ refers to the predicted probability of pixel *i* belonging to the foreground class, $g_{i}$ is the ground truth label for pixel *i*, which is 1 for foreground and 0 for background, and *N* is the number of total pixels in the predicted and ground truth images.

Tyversky loss $L_{Tyv}$ is a generalization of the Dice loss, providing more flexibility in handling false positives and false negatives:

$$\begin{array}{c} L_{Tyv}=1-\frac{\sum_{i=1}^{N}p_{i}g_{i}+\epsilon }{\sum_{i=1}^{N}p_{i}g_{i}+\alpha \sum_{i=1}^{N}p_{i}\left(1-g_{i}\right)+\beta \sum_{i=1}^{N}\left(1-p_{i}\right)g_{i}+\epsilon }, \end{array}$$

where $p_{i}$ is the predicted probability of pixel *i* belonging to the foreground class, $g_{i}$ is the ground truth label for pixel *i*, which is 1 for foreground and 0 for background, α and β are weights to control the relative importance of false negatives and false positives, respectively, and ϵ is a small constant (like $1\times 10^{-5}$) added for numerical stability; *N* is the number of pixels in the predicted and ground truth images.

The accuracy metric is based on the accuracy calculated from segmentation mask as the measure of performance.

### 4.3. LD (Lane Detection)

For lane detection, the lane area was derived by treating it as a branch of segmentation, in a similar way as DAS. In recent lane detection studies UFLD [18] and CLRNet [57], row anchor-based approaches have demonstrated superior accuracy performance. However, as these approaches necessitate additional backbones, subnets, and post-processing steps, they result in increased latency. To minimize latency and leverage the potential of the existing backbone and subnet, a segmentation-based technology was employed in this study.

Similar to the case of DAS (drivable area segmentation), the accuracy metric for this task is based on the accuracy calculated from the segmentation mask, serving as the performance measure.

### 4.4. DE (Depth Estimation)

A subnet was designed based on MonoDepth [58], which is an FPN based depth estimation technology.

SigLoss (scale-invariant gradient loss) $L_{Sig}$ and BerhuLoss $L_{Berhu}$ [59] were employed as loss functions, with the more effective loss function value being selected and utilized based on experimental outcomes.

SigLoss $L_{Sig}$ is used to ensure that the estimated depth maps maintain correct local structures and gradients relative to the ground truth. This loss is particularly useful for preserving edge information and relative depth differences, regardless of the absolute scale:

(5) $$\begin{array}{c} L_{Sig}=\frac{1}{N}\sum_{i=1}^{N}{\left(\Delta d_{i}^{pred}-\Delta d_{i}^{true}\right)}^{2} \end{array}$$

where *N* is the total number of pixels, and $\Delta d_{i}^{pred}$ and $\Delta d_{i}^{true}$ are the gradients (spatial derivatives) of the predicted and true depth values at pixel *i*, respectively. The sum of squared differences in gradients across all pixels is calculated and normalized by the number of pixels.

BerhuLoss $L_{Berhu}$ is a loss function that combines the properties of *L*1 for small errors and *L*2 losses for larger errors:

(6) $$\begin{array}{c} L_{Berhu}=\left\{\begin{array}{cc} |y-\hat{y}| & \mathrm{for}\;|y-\hat{y}|\leq c\\ \frac{{\left(y-\hat{y}\right)}^{2}+c^{2}}{2c} & \mathrm{for}\;|y-\hat{y}|>c \end{array}\right. \end{array}$$

where *y* is the true value (in this case, the true depth), $\hat{y}$ is the predicted value (the estimated depth), and *c* is a threshold that determines the switch between the *L*1-like and *L*2-like behavior.

For accuracy metrics, the *REL* (absolute relative error) values for depth information of each pixel were utilized.

(7) $$\begin{array}{c} REL=\frac{1}{N}\sum_{i=1}^{N}\frac{|d_{i}^{pred}-d_{i}^{true}|}{d_{i}^{true}}, \end{array}$$

where *N* is the total number of pixels (or points) for which the depth is being estimated, $d_{i}^{pred}$ is the predicted depth for the *i*th pixel (or point), and $d_{i}^{true}$ the ground truth depth for the *i*th pixel (or point).

## 5. Simulation Results

The evaluation of various tasks are conducted under the umbrella of MTL, analyzing them both individually and in integrated configurations, ranging from single-task scenarios to combinations of up to four tasks. Furthermore, the results were benchmarked against previous MTL techniques, notably YOLOP [20] and HybridNet [19]. This comparison was extended to traditional OD (object detection) strategies, such as RetinaNet [8], LD (lane detection) methods such as UFLD [18] and CLRNet [57], DE (depth estimation) such as DepthFormer, and DAS (drivable area segmentation) approaches utilizing architectures like FPN, PFPN, BiFPN, and Transformer (SegFormer) mentioned in Section 3.

To assess the MDO algorithm, the optimal multi-task set $T_{m}^{*}$, SBM *B*, and TSM *S* are determined within parameters exceeding the targeted accuracy of 95 % for LD and DAS, surpassing the targeted mAP of 0.80 for OD, and falling below the targeted absolute REL (relative error) of 0.06 for DE. Subsequently, the accuracy and latency performances of these optimized sets are evaluated.

The BDD 100K and the KITTI datasets in [60,61] were employed for training and evaluation. Specifically, whereas the BDD 100K dataset contains labels for DAS, such labels are absent in the KITTI dataset. To address this, SSL (semi-supervised learning) was applied to the KITTI dataset for additional training. More precisely, a pseudo label was created using an InterImage model [51] pretrained on Cityscapes [62], which was then utilized to apply semi-supervised learning for the DAS task. Experiments were executed using implementations in TensorFlow, facilitated by an NVIDIA GPU equipped with a 2-way 4090 architecture. A piecewise constant decay strategy was adopted for the learning rate schedule. Model performances were assessed across a span of 50 epochs, with the most optimal outcome within this range being chosen for further analysis. The AdamW was employed as the optimizer algorithm. Each task-specific loss value in MTL was trained through the summation of loss values derived in Equation (3), using the functions mentioned in Section 4. The weight $\lambda_{i}$ for each loss function was set to 2 for object detection and maintained at 1 for the remaining tasks.

**Figure 3:**
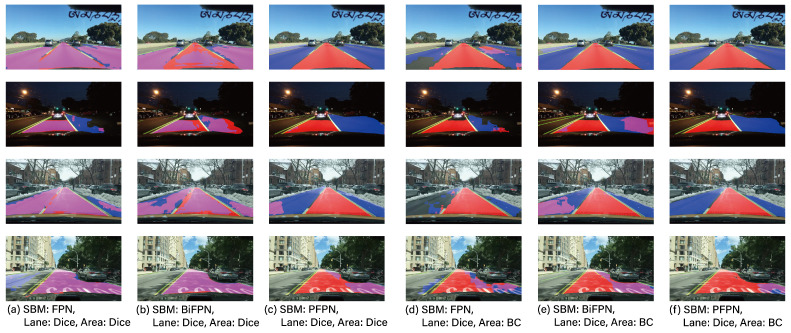
The example results of two tasks.

**Figure 4:**
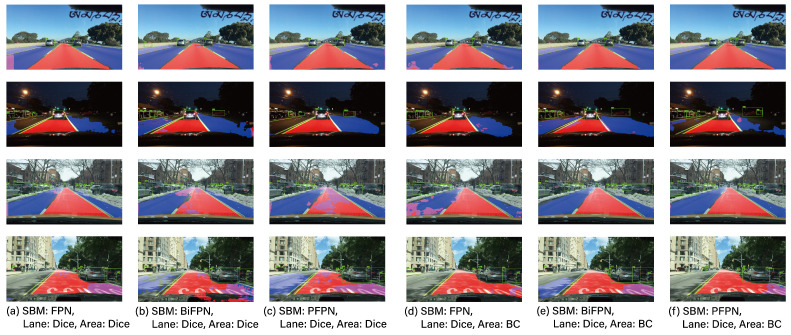
The example results of three tasks.

**Figure 5:**
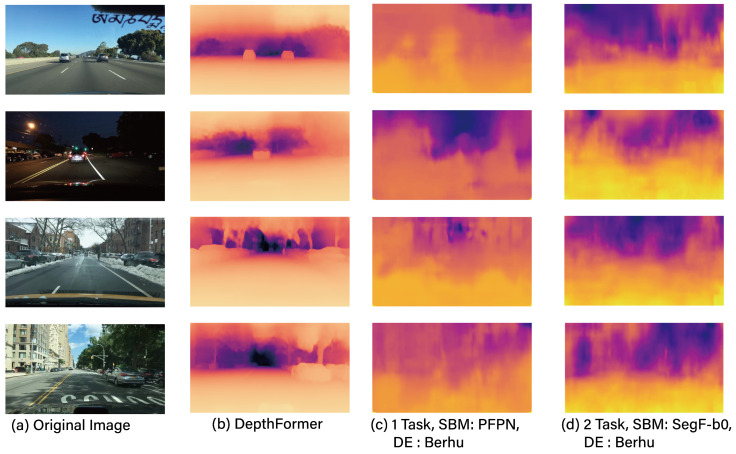
The example results for depth estimation.

For the performance analysis of each task (OD, LD, DAS, and DE) in MTL, experimental groups were set up with a dedicated 1-task model, a 2-task model (DAS + LD, DAS + OD, OD + LD, DE + DAS) and a 3-task model (OD + LD + DAS), and their respective performances were compared. Table 2 shows the performance of DAS, Table 3 presents the performance of OD, Table 4 illustrates the performance of LD, and Table 5 presents the performance of DE. Additionally, Figure 3, Figure 4 and Figure 5 provide visual examples of the application of these 2-task and 3-task scenarios.

Based on the results of all experimental groups presented in Table 3, Table 4 and Table 5, it is evident that the applications of depth estimation are insufficient for ensuring safe autonomous driving. As evidenced by Table 2 and Table 5, the results for the 2-task (DAS + DE) setup indicate that DAS does not meet its target accuracy of 95%, and, similarly, DE falls short of the target REL of 0.06. In contrast, other experimental sets excluding DE, such as 1-task, as well as 2-task and 3-task configurations, generally satisfy their target performance.

This can be attributed to the fact that tasks such as DAS and LD have high ITC, leading to their backbone weights being trained to exhibit similar distributions, which in turn enhances their collective performance. Conversely, tasks like OD and DE have less ITC, resulting in them being trained with different backbone weight distributions, ultimately leading to a mutual degradation in performance.

Therefore, it can be inferred that tasks for multi-task learning can be readily trained to assist each other in improving accuracy, whereas some MTS configurations may not offer such benefits. Consequently, constructing an MTS with such complementary tasks is instrumental in enhancing the safety of autonomous driving. Additionally, the multi-task learning examples presented in this study reveal that operating with only three tasks—OD, LD, and DAS—excluding DE, provides a more secure and efficient approach to securing an autonomous driving image recognition model.

Moreover, although the backbone models generally exhibit similar performance, it is noteworthy that in the 3-task configuration, BiFPN demonstrates the best performance. This surpasses even the Transformer-based SegFormer and PFPN, which have the highest number of parameters. This suggests that for the KITTI dataset of DAS, OD, and LD tasks, the BiFPN model, with fewer parameters than the SegFormer and PFPN, is less prone to overfitting and offers better generalization effects.

Furthermore, it can be observed that this parallel processing approach in multi-task learning offers significant advantages in terms of latency. As indicated in Table 2, Table 3 and Table 4, for the 2-task configuration, there is an approximate 50% reduction in latency, while for the 3-task setup, the latency reduction effect can reach around 60% compared to the individual task learning.

Table 6 selectively compares the system load of 3-task learning in experimental sets that meet the performance requirements of each task. The results presented are after the application of step 3 of the MDO algorithm, which is TensorFlow-Lite based FP16 quantization [63]. The rationale for employing FP16 quantization is that, compared to other quantization techniques, it incurs the least accuracy loss and can halve the memory size, while having no impact on operational latency [5]. This demonstrates that the application of the MDO algorithm can achieve optimal adjustments suitable for autonomous driving in terms of accuracy, latency, and memory size.

Next, let us delve into an analysis of the loss functions utilized for the DAS and LD tasks, as presented in Table 2 and Table 4. Traditionally, in image segmentation problems, the binary cross-entropy function is predominantly employed. However, to address class imbalance issues, the Dice and Tversky functions are utilized [52]. As can be discerned from Figure 3 and Figure 4, the proportion of the foreground area is relatively small compared to the entirety of the image. Consequently, based on the results presented in Table 2 and Table 4, the proposed DAS and LD techniques demonstrate superior performance with the Dice function rather than the BC. Notably, conventional methods such as YOLOP and HybridNet also employ a similar Tversky function as their loss function. Given this context, it would be prudent to utilize the Dice or Tversky loss functions in autonomous driving applications, taking into account the dimensions of the foreground areas.

From the aforementioned results, it can be observed that the REL of the dedicated model for the DE task, i.e., DepthFormer, demonstrates superior performance compared to other experimental groups. It is imperative to note that even the singular task configuration showcases a suboptimal REL metric, which further deteriorates when subjected to multi-task operational paradigms, as exemplified by the 2-task model. From the foregoing analysis, it becomes apparent that implementing depth estimation via MTL frameworks is suboptimal, given the intrinsically low degree of ITC between the DE task and other associated tasks. Furthermore, as elucidated in [64], the particular task under consideration is not optimally aligned for integration within MTL frameworks. This is attributed to its heightened dependence on supplementary operations external to the backbone structure (e.g., T-Net), rather than on the primary backbone task. This aspect categorically renders it as a technically misaligned group for MTL applications. Additionally, an examination of the performance metrics associated with the 2-task (DAS+DE) configuration, as detailed in Table 2, reveals a concurrent degradation in the performance of DAS, another task intricately connected with DE. This observation underscores the inappropriateness of sharing a common backbone between the DE and DAS tasks. However, as depicted in Table 6, the dedicated model approach, exemplified by DepthFormer, necessitates the utilization of additional parameters compared to the MTL methodology, resulting in augmented costs for securing the requisite resources. Consequently, a comprehensive consideration of both the additional resource costs and accuracy is imperative when determining the application of MTL to DE.

## 6. Conclusions

This study explores MTL for maximizing the efficiency of various image recognition tasks performed in autonomous driving, considering the task characteristics and the given hardware conditions. Additionally, MDO algorithm, an optimal configuration algorithm for this purpose, is proposed. The MDO algorithm targets drivable area segmentation, object detection, lane detection, and depth estimation as the tasks for recognition, and is comprised of three stages: minimizing latency, maximizing accuracy, and minimizing size. Through the MDO algorithm, an optimal neural network design including the backbone and loss functions is achieved. Additional training based on the SSL led to improvements in all aspects—accuracy, latency, and size—compared to traditional single-task methods and existing three-task learning approaches. The experimental results reveal that integrated accuracy performance is crucial in the configuration and optimization of MTL, and this integrated accuracy is determined by the ITC. Considering these characteristics, it was proven important to design multi-task sets comprising tasks with high ITC. The proposed MDO algorithm facilitated approximately a 12% improvement in object detection mAP performance, a 15% enhancement in lane detection ACC, and a 27% reduction in execution time. Additionally, it has been found that depth estimation has a low ITC with tasks such as drivable area segmentation, object detection, and lane detection. Forming a multi-task set with these tasks could potentially lead to mutual performance degradation. Therefore, to achieve stable performance in depth estimation, it is concluded that it should be implemented either through a dedicated independent neural network or conducted using additional sensors like lidar. In the future, research is planned to extend MTL based on sensor fusion, not only through single-sensor inputs from cameras but also incorporating inputs from lidar sensors. This expansion aims to enhance the currently limited performance of depth estimation. Additionally, the scope of research will be extended to encompass the entire process of perception, decision-making, and control in autonomous driving, achieving an end-to-end learning approach. This will facilitate both horizontally and vertically integrated optimization in the field.

## Figures and Tables

**Table 1:** Parameters for describing MDO.

Notation	Meaning
$T_{t}$	Total task set of image recognition for autonomous driving
$T_{m}$	Task set to which MTL is applied, $T_{m}\subset T_{t}$
$t_{i}$	Individual task to which MTL is applied, $t_{i}\in T_{m}$
$T_{m^{c}}$	Task sets to which MTL is not applied, $T_{m^{c}}=T_{t}\setminus T_{m}$
$t_{j}$	Individual task to which MTL is not applied, $t_{j}\in T_{m^{c}}$
$LM(t_{i},B)$	Latency of task $t_{i}$ with SBM B
$LI(t_{j})$	Latency of task $t_{j}$
$Acc(t_{i})$	Accuracy of task $t_{i}$
$Size(t_{i},W_{B},W_{S})$	Size of SBM $W_{B}$ and TSM $W_{S}$ for task $t_{i}$
$\gamma_{i}$	Accuracy threshold of task $t_{i}$
$\theta_{i}$	Latency threshold of task $t_{i}$
$L(t_{i})$	Loss function of task $t_{i}$ for training

**Table 2:** Performance results of MTL for drivable area segmentation task.

	**1 Task (DAS)**						
Model	UNet	FPN	BiFPN	PFPN	TRN		
Best Loss	Dice	Dice	Dice	Dice	Dice		
ACC	0.93	0.93	0.95	0.95	0.95		
Perf. Req.	X	X	O	O	O		
Lat (ms)	24	24	25	25	60		
	**2 Tasks (DAS + LD)**						
Model	UNet	FPN	BiFPN	PFPN	TRN		
Best Loss	Dice	Dice	Dice	Dice	Dice		
ACC	0.92	0.94	0.95	0.95	0.95		
Perf. Req.	X	X	O	O	O		
Lat (ms)	26	26	27	27	62		
	**2 Tasks (DAS + DE)**						
Model	UNet	FPN	BiFPN	PFPN	TRN		
Best Loss	Dice	Dice	Dice	Dice	Dice		
ACC	0.59	0.62	0.66	0.68	0.71		
Perf. Req.	X	X	X	X	X		
Lat (ms)	31	31	32	32	61		
	**2 Tasks (DAS + OD)**						
Model	UNet	FPN	BiFPN	PFPN	TRN		
Best Loss	Dice	Dice	Dice	Dice	Dice		
ACC	0.92	0.94	0.95	0.95	0.92		
Perf. Req.	X	X	O	O	X		
Lat (ms)	26	26	27	28	63		
	**3 Tasks (DAS + LD + OD)**					**Prev 3 Tasks**	
Model	UNet	FPN	BiFPN	PFPN	TRN	YOLOP	HybridN
Best Loss	Dice	Dice	Dice	Dice	Dice	Tyv	Tyv
ACC	0.90	0.91	0.95	0.95	0.90	0.97	0.91
Perf. Req.	X	X	O	O	X	O	X
Lat (ms)	30	30	31	32	72	44	61

**Table 3:** Performance results of MTL for object detection task.

	**1 Task**						
Metric	UNet	FPN	BiFPN	PFPN	TRN		
mAP	0.83	0.88	0.88	0.88	0.88		
Lat (ms)	26	25	25	25	61		
	**2 Tasks (OD + DAS)**						
Metric	UNet	FPN	BiFPN	PFPN	TRN		
mAP	0.82	0.85	0.87	0.86	0.88		
Lat (ms)	26	26	27	28	63		
	**2 Tasks (OD + LD)**						
Metric	UNet	FPN	BiFPN	PFPN	TRN		
mAP	0.82	0.86	0.87	0.86	0.87		
Lat (ms)	26	26	26	28	62		
	**3 Tasks (OD + LD + DAS)**					**Prev 3 Tasks**	
Metric	UNet	FPN	BiFPN	PFPN	TRN	YOLOP	HybridN
mAP	0.81	0.86	0.87	0.87	0.85	0.76	0.77
Perf. Req.	O	O	O	O	O	X	X
Lat (ms)	30	30	31	32	72	44	61

**Table 4:** Performance results of MTL for lane detection task.

	**1 Model**		**1 Task (LD)**				
Metric	UFLD	CLRNet	UNet	FPN	BiFPN	PFPN	TRN
Best Loss	Dice	Dice	Dice	Dice	Dice	Dice	Dice
mAP	0.98	0.99	0.95	0.97	0.98	0.97	0.98
Lat (ms)	10	11	24	24	25	25	60
	**2 Tasks (LD + DAS)**						
Model	UNet	FPN	BiFPN	PFPN	TRN		
Best Loss	Dice	Dice	Dice	Dice	Dice		
ACC	0.96	0.96	0.98	0.97	0.95		
Lat (ms)	26	26	27	27	62		
	**2 Tasks (LD + OD)**						
Model	UNet	FPN	BiFPN	PFPN	TRN		
Best Loss	Dice	Dice	Dice	Dice	Dice		
ACC	0.93	0.96	0.98	0.95	0.90		
Lat (ms)	26	26	26	28	62		
	**3 Tasks (LD + DAS + OD)**					**Prev 3 Tasks**	
Model	UNet	FPN	BiFPN	PFPN	TRN	YOLOP	HybridN
Best Loss	Dice	Dice	Dice	Dice	Dice	Tyv	Tyv
ACC	0.89	0.94	0.98	0.98	0.86	0.70	0.85
Perf. Req.	X	X	O	O	X	X	X
Lat (ms)	30	30	31	32	72	44	61

**Table 5:** Performance results of MTL for depth estimation task.

	**1 Model**	**1 Task (DE)**				
Metric	DepthF	UNet	FPN	BiFPN	PFPN	TRN
Best Loss	Berhu	Berhu	Berhu	Berhu	Berhu	Berhu
REL	0.0528	0.122	0.098	0.096	0.095	0.074
Perf. Req.	O	X	X	X	X	X
Lat (ms)	37	24	24	25	55	60
	**2 Tasks (DE + DAS)**					
Model	UNet	FPN	BiFPN	PFPN	TRN	
Best Loss	Berhu	Berhu	Berhu	Berhu	Berhu	
REL	0.182	0.167	0.155	0.154	0.116	
Perf. Req.	X	X	X	X	X	
Lat (ms)	31	31	32	32	61	

**Table 6:** System load of triple-task learning after implementing step 3 of the MDO algorithm (target accuracy of 95% for LD, DAS, and target mAP of 0.80 for OD).

	3 Tasks (DAS + OD + LD)					Prev 3 Tasks	
Model	UNet	FPN	BiFPN	PFPN	TRN	YOLOP	HybridN
Perf. Req.	X	X	O	O	X	X	X
Parameters (Mega)	4.3	3.8	3.5	4.9	3.8	7.9	12.8
Base (MB)	522	456	425	597	466	91	54
Compressed (MB)	97	76	70	99	77	-	-
Lat (ms)	30	30	31	32	72	44	61

## Data Availability

Data are contained within the article.
